# Functional Insights into the RCC1 Gene Family and UVR8-Mediated Regulation of Anthocyanin Biosynthesis in Grapevine

**DOI:** 10.3390/plants15111709

**Published:** 2026-05-31

**Authors:** Hamza Ali, Qian Xu, Jiahao Fu, Wen Zhao, Lu Bian, Yiqing Xu, Qiqi Wang, Yifei Zhang, Xiaoqiao Zhou, Xiaoxuan Jia, Yi Tong, Yan Xu, Tengfei Xu

**Affiliations:** State Key Laboratory for Crop Stress Resistance and High-Efficiency Production, College of Horticulture, Northwest A&F University, Yangling 712100, China; hamzaali@nwafu.edu.cn (H.A.);

**Keywords:** *Vitis vinifera*, fruit crops, Regulator of Chromosome Condensation 1 (RCC1), gene family analysis, abiotic stresses, *UVR8*

## Abstract

Grapevine (*Vitis vinifera* L.) is a key fruit crop affected by abiotic stresses such as salinity, drought, and temperature extremes. The Regulator of Chromosome Condensation 1 (RCC1) family, involved in regulating Ran GTPase activation, nucleocytoplasmic transport, and chromatin organization, has not been comprehensively characterized in grapevine. In this study, we identified 26 *VvRCC1* genes, which were classified into five phylogenetic groups, and analyzed their distribution across the grapevine genome. These genes exhibited significant diversity in physicochemical properties, suggesting functional divergence. Expression profiling revealed distinct spatiotemporal patterns, indicating roles in both vegetative growth and reproductive development. Notably, several VvRCC1 genes showed differential responses to salinity, drought, and heat stress. Importantly, *VvRCC1-17*, identified as *UVR8*, was shown to regulate anthocyanin biosynthesis under UV-B exposure. OE-*VvUVR8* transgenic grape calli exhibited increased anthocyanin accumulation, reflected in a distinct red coloration compared to wild-type calli. This finding links *UVR8* to light signaling and pigmentation pathways in grapevine, providing the first comprehensive analysis of the RCC1 gene family in grapevine and highlighting *VvRCC1-17* (*UVR8*) as a key regulator of UV-induced anthocyanin biosynthesis, offering insights into the molecular mechanisms of stress adaptation and pigment regulation.

## 1. Introduction

Plants are constantly faced with several environmental stresses that seriously affect their growth, development, and productivity. Adverse environmental conditions like salinity, temperature fluctuations, and drought conditions significantly influence the plants yield and survival ability [1]. They have evolutionary regulatory networks that influence the plant’s signaling, expression of stress-related genes, and remodeling of the chromatin structures to cope with these adverse conditions, ensuring its survival [2,3]. Regulator of chromosome condensation 1 (RCC1) protein is one of the integral parts of such regulatory mechanisms. It consists of an RCC1 domain and is conserved among plants [4]. RCC1 genes ensure the integrity of the genome and are identified for signaling mechanisms in plants under different environmental conditions [5].

In eukaryotic cells, RCC1 functions as the principal chromatin-associated guanine nucleotide exchange factor (GEF) for the small GTPase Ran, a master regulator of nucleocytoplasmic trafficking and cell-cycle progression. By catalyzing the exchange of GDP for GTP on Ran, RCC1 establishes a steep concentration gradient of Ran-GTP across the nuclear envelope, high within the nucleus and low in the cytoplasm [6]. This gradient constitutes the thermodynamic driving force and spatial cue for nucleocytoplasmic transport mediated by the importin-β family of nuclear transport receptors (karyopherins), which bind cargo proteins in the cytoplasm and release them upon encountering Ran-GTP in the nucleoplasm [6,7]. During mitosis, chromatin-tethered RCC1 maintains local Ran-GTP enrichment, which drives the assembly of microtubule spindle fibers, ensures proper kinetochore attachment, and safeguards chromatin structural integrity through regulated nuclear envelope dynamics [6]. In plants, the emerging roles of small GTPases and their interactomes in stress responses further underscore the importance of Ran-mediated signaling [7]. Beyond this canonical GEF activity, the RCC1 family has undergone substantial diversification in the plant lineage, with individual members acquiring specialized functions in light signal transduction, hormonal crosstalk, and abiotic stress adaptation while retaining the conserved seven-bladed β-propeller domain architecture [5,8]. The first plant RCC1 member to be functionally characterized as a non-canonical photoreceptor was *UV RESISTANCE LOCUS 8* (*UVR8*) in *Arabidopsis thaliana*; upon UV-B absorption, UVR8 undergoes instantaneous conversion from a homodimer to a monomer and interacts with CONSTITUTIVELY PHOTOMORPHOGENIC 1 (COP1) to direct photomorphogenic development [9]. Additionally, RCC1 proteins mediate environmental stress responses; for instance, in rice (*Oryza sativa*), OsRCC1 contributes to cold and dehydration tolerance by modulating reactive oxygen species (ROS) homeostasis and abscisic acid (ABA) signaling [10], whereas in soybean (*Glycine max*), GmTCF1a enhances freezing tolerance [11]. Collectively, these findings establish RCC1 proteins as pivotal integrators of the plant molecular network, enabling adaptation to changing environmental conditions. Despite this growing body of evidence in model and herbaceous species, a genome-wide characterization of RCC1 genes in woody perennials, particularly economically important crops like grapevine, remains largely unexplored.

Grapevine is one of the most widely cultivated fruit crops globally, valued not only for fruit production but also for its role in winemaking [12]. However, its productivity and fruit quality are significantly impacted by abiotic stresses such as drought, salinity, extreme temperatures, and water scarcity, limiting its cultivation in many regions [13]. Recent research has identified various transcription factor families and signaling genes in grapevine that are activated in response to abiotic stresses. However, the grapevine RCC1 gene family has not been previously identified or functionally analyzed. There remains a critical gap in our understanding of the molecular mechanisms underlying grapevine stress tolerance and growth regulation. Understanding the role of RCC1 genes in stress adaptation, genome maintenance, and pathway regulation in grapevine is essential. Moreover, a comprehensive genome-wide assessment of RCC1 genes across various species could reveal how these genes have evolved to address specific environmental and physiological challenges. Grapevine, with its broad spectrum of stress tolerance, serves as an ideal model for such studies. The present study focuses on the genome-wide identification and characterization of the RCC1 gene family in grapevine, aiming to provide insights into their functional roles in stress responses. Additionally, the study explores how *UVR8*, a key member of the RCC1 family, regulates anthocyanin biosynthesis under UV exposure. This will enhance our understanding of the full scope of RCC1 gene functions and could provide novel targets for improving stress resilience in grapevine.

## 2. Results

### 2.1. Identification, Physicochemical Properties, and Chromosomal Distribution of VvRCC1 Genes

After removing redundant and incomplete sequences and verifying domains, a total of 26 RCC1s were identified in the whole *V. vinifera* genome, designated as *VvRCC1-1* to *VvRCC1-26* according to their chromosomal location. The physicochemical properties of *VvRCC1s* differ greatly. The size of the encoded proteins ranged from 186 (*VvRCC1-15*) to 1129 (*VvRCC1-24*) amino acids, and their predicted molecular mass ranged from 19,713.48 (*VvRCC1-15*) to 122,282.54 Da (*VvRCC1-24*). The isoelectric points of the predicted proteins ranged from 5.12 (*VvRCC1-5*) to 9.56 (*VvRCC1-21*), with an average pI value of 7.36. The genes with negative GRAVY values are hydrophilic in nature, while those with positive GRAVY values are hydrophobic in nature. The majority of the VvRCC1s had negative GRAVY values, except for *VvRCC1-7*, *VvRCC1-15*, *VvRCC1-16*, and *VvRCC1-21*. *VvRCC1-23* showed the least GRAVY value of −0.649, while the *VvRCC1-21* had the highest GRAVY value of 0.333. The average GRAVY value of these VvRCC1’s was −0.232. Subcellular localization prediction showed that *VvRCC1-5* was present in both mitochondria and the nucleus. The *VvRCC1-15* was present in the cell wall and nucleus, while the rest of the VvRCC1’s were present in the nucleus. The detailed properties of *VvRCC1*’s are shown in Table 1.

**Figure 1:**
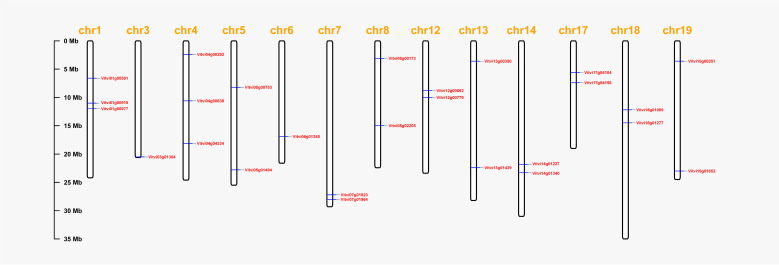
Chromosomal distribution of the identified RCC1 gene family members in *V. vinifera*. The size of each chromosome is expressed by its relative length. The chromosome numbers are shown on the top of each chromosome. The scale bar on the left indicates the chromosome lengths in megabases (Mb).

The 26 *VvRCC1* genes were unevenly distributed across 13 chromosomes of the *V. vinifera* genome (Figure 1). The chromosomal distribution analysis revealed that VvRCC1 genes were unevenly distributed across 13 chromosomes of *V. vinifera*, specifically chromosomes 1, 3, 4, 5, 6, 7, 8, 12, 13, 14, 17, 18, and 19. Among these, Chromosomes 1 and 4 each harbor the largest clusters, containing three RCC1 loci per chromosome (Chr1: *Vitvi01g00591*, *Vitvi01g00915*, *Vitvi01g00977*; Chr4: *Vitvi04g00292*, *Vitvi04g00838*, *Vitvi04g04334*). Chromosomes 5, 7, 8, 12, 13, 14, 17, 18, and 19 each contain two RCC1 members (Chr5: *Vitvi05g00753*, *Vitvi05g01494*; Chr7: *Vitvi07g01923*, *Vitvi07g01964*; Chr8: *Vitvi08g00173*, *Vitvi08g02205*; Chr12: *Vitvi12g00662*, *Vitvi12g00779*; Chr13: *Vitvi13g00350*, *Vitvi13g01429*; Chr14: *Vitvi14g01227*, *Vitvi14g01340*; Chr17: *Vitvi17g04104*, *Vitvi17g04156*; Chr18: *Vitvi18g01069*, *Vitvi18g01277*; Chr19: *Vitvi19g00251*, *Vitvi19g01652*). In contrast, chromosomes 3 and 6 each carry a single RCC1 locus (Chr3: *Vitvi03g01304*; Chr6: *Vitvi06g01245*). No VvRCC1 genes were detected on chromosomes 2, 9, 10, 11, 15, and 16. These non-random patterns, notably the multi-gene clusters on Chr1 and Chr4 and the several two-member chromosomes, are consistent with a history of segmental and local duplication driving family expansion. The genomic positions correspond with Table 1, where CDS lengths span from 561 to 4335 bp and predicted protein lengths range from 186 to 1129 aa; the dispersed yet clustered arrangement across the genome implies retention of paralogs with potentially divergent regulatory or functional roles in chromatin regulation, nucleocytoplasmic transport and cell-cycle control. This chromosomal mapping provides physically anchored loci for marker-assisted selection and genome editing strategies, while the identification of stress-responsive clusters offers candidate targets for improving grapevine resilience to environmental stresses.

### 2.2. Classification and Phylogenetic Tree of VvRCC1

**Figure 2:**
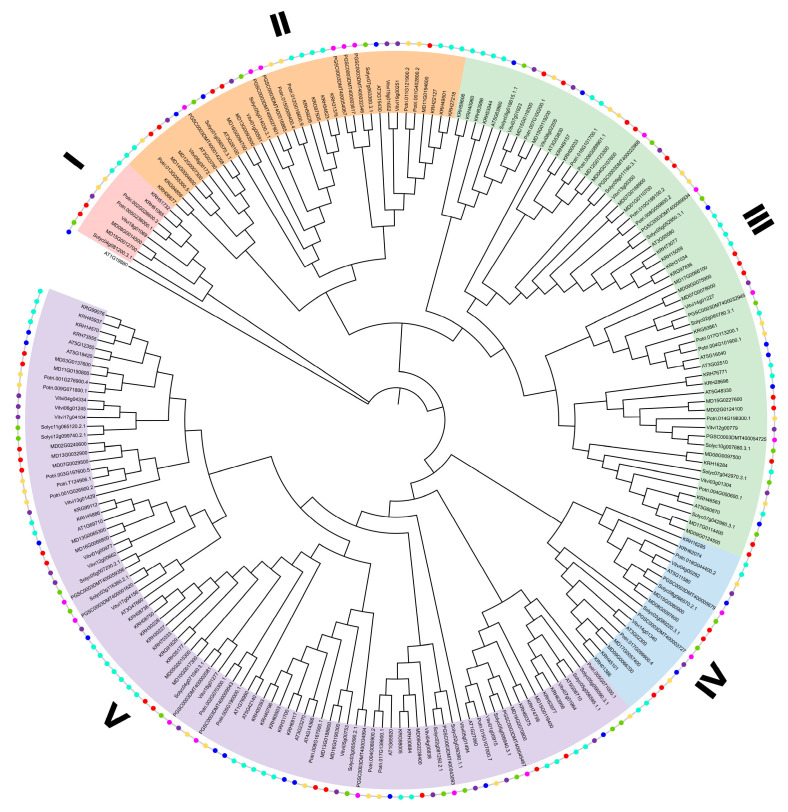
Phylogenetic relationships of RCC1 proteins from seven plant species. Phylogenetic trees were constructed using the RCC1 protein sequences. The members of the RCC1 family are divided into four groups: I, II, III, IV and V. Each distinct clade is distinguished by color. RCC1 proteins of different plant species: 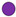 *Vitis vinifera*, 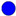 *Arabidopsis thaliana*, 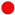 *Malus domestica*, 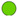 *Solanum lycopersicum*, 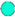 *Glycine max*, 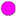 *Solanum tuberosum*, and 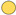 *Populus trichocarpa*.

To elucidate the phylogenetic associations among RCC1 proteins throughout distinct plant lineages, we generated a neighbor-joining based phylogeny on the basis of sequence alignment data of RCC1 proteins from grapevine (*Vitis vinifera*), *Arabidopsis thaliana*, apple (*Malus domestica*), Soybean (*Glycine max*), tomato (*Solanum lycopersicum*), poplar (*Populus trichocarpa*) and Potato (*Solanum tuberosum*) (Figure 2 and Table S1). The results indicated that RCC1 homologs from these plant lineages were categorized into five clades (I to V). Notably, groups I, II, III, IV and V consisted of 1, 4, 5, 3 and 13 VvRCC1 members, respectively, suggesting unequal arrangement of *Vitis* RCC1 genes among distinct cladistic lineages. Moreover, it showed distinct phylogenetic relationships, with VvRCC1 genes grouping into diverse major clades. Group, I contained one member, *Vitvi18g01069*. Group II contained four members: *Vitvi08g00173*, *Vitvi01g00591*, *Vitvi19g01652*, and *Vitvi19g00251*. Group III contained six members: *Vitvi07g01923*, *Vitvi08g02205*, *Vitvi13g00350*, *Vitvi14g01227*, *Vitvi12g00779*, and *Vitvi03g01304*. Group IV contained two members: *Vitvi04g00292* and *Vitvi14g01340*. Group V contained thirteen members: *Vitvi07g01964*, *Vitvi01g00915*, *Vitvi05g01494*, *Vitvi04g00838*, *Vitvi05g00753*, *Vitvi18g01277*, *Vitvi17g04156*, *Vitvi12g00662*, *Vitvi01g00977*, *Vitvi13g01429*, *Vitvi17g04104*, *Vitvi06g01245*, and *Vitvi04g04334*. Hence, the highest number of *Vitis vinifera* RCC1 genes were present in clade V, while clade I comprised only one RCC1 family member.

Clade V showed stronger revolutionary relationships with RCC1 genes from tomato and potato, indicating a comparatively stable phylogenetic lineage among these members of the *Solanaceae* spp. Relatively, the remaining VvRCC1 genes were classified distinctly with homologous RCC1 genes from *P. trichocarpa* and *A. thaliana*, suggesting extensive preservation of RCC1 genes across genetically divergent plant species. These grouping patterns reveal that both specie-level divergence and duplication events across genes have facilitated the lineage expansion and functional divergence of the RCC1 genes. Remarkably, the Clade V comprised 13 VvRCC1 genes, accounting for nearly half of the VvRCC1 genes in grapes, suggesting a clade-dependent expansion. In contrast, the occurrence of only one VvRCC1 gene in Clade I suggests a stable evolutionary position, possibly linked to vital cellular roles. Overall, these results demonstrate that the RCC1 gene family in *V. vinifera* has experienced both species-specific diversification and cross-species conservation, providing a solid evolutionary framework for subsequent analyses of gene structure, conserved motifs, tissue-specific expression, and regulation under abiotic stress conditions (Figure 2).

### 2.3. Structure Analysis of VvRCC1s

**Figure 3:**
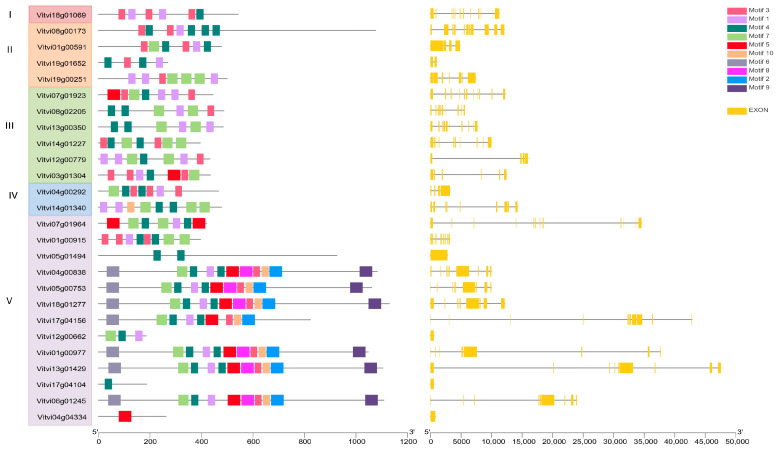
Gene structure and conserved motif composition of RCC1 gene family members in *V. vinifera*. The prediction identified 10 conserved motifs within VvRCC1 proteins, with each box representing a specific motif. Diverse protein domains are illustrated by bars of distinct colors. The structure of *VvRCC1* genes is depicted with yellow blocks for exons and gray lines for introns.

To investigate the structural characteristics of *VvRCC1*’s, we analyzed their exon–intron structures (Figure 3). We found that there was significant variation in exon-intron structures among the RCC1 family members. Whereas the genes that were present in the same phylogenetic group (Groups I–V) had similar structures with almost same exon lengths. The VvRCC1 proteins had a total of 10 motifs, i.e., Motif 1–10. Majority of the VvRCC1 family members had multiple motifs except a few indicating the rearrangement of partial motif loss. We found that the members of the group I have fewer motifs, where most RCC1 members consisting of Motifs 1–4. Group II consist of a complex motif with more structural diversity, mostly made up of motifs 1–7. Similarly Group III showed the most variability in the motif composition. It has a total of 10 motifs present in it thus indicating its functional versatility. Members of the Group IV were moderately conserved, mostly made up of motifs 1–6 in a similar arrangement. Likewise, members of the Group V showed highly conserved motif patterns having almost identical motif combinations and structures, showing strong evolutionary conservation. Overall, most of the VvRCC1 members shared the Motifs 1–7 while Motifs 8–10 was observed in a few genes. 

### 2.4. Collinearity Analysis of VvRCC1s

**Figure 4:**
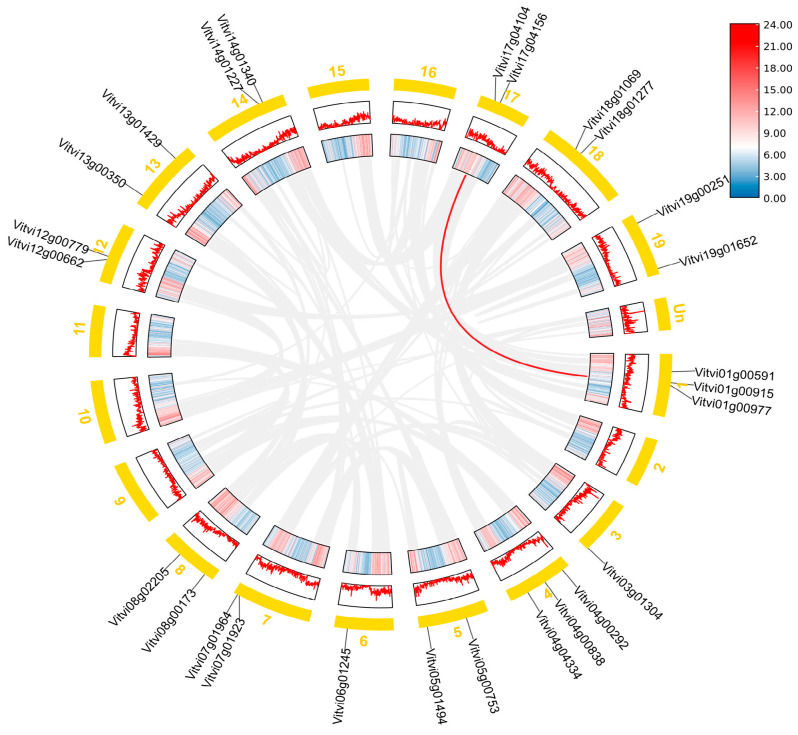
Intra-group collinearity analysis of the *VvRCC1* genes in *V. vinifera*. Intraspecies collinearity analysis of *VvRCC1s* with the *V. vinifera* genome. Gray lines represent all syntenic blocks, while red lines indicate the segmental replication gene pairs. The outermost yellow circle represents the 20 chromosomes of *V. vinifera* with gene locations labeled along each chromosome, and the inner two circles using both heatmap and line tracks represent the gene density on each chromosome.

Intraspecies collinearity analysis indicated several segmental duplication events among *VvRCC1* members, primarily located on chromosomes 5, 13, and 17, suggesting that segmental duplication has been the major driving force behind the expansion of the *RCC1* gene family in grapevine. According to the strong collinearity relationships observed among duplicated gene pairs, it is likely that they have emerged from a state of purifying selection, signifying that these genes have retained a conserved functional role during the course of evolution. In contrast, segmental duplication was identified as the dominant expansion mechanism, with one duplicated gene pair (*Vitvi01g00977* and *Vitvi17g04156*) located on chromosomes 1 and 17, respectively. The heat map shown below appears to show differential expressions of *VvRCC1* genes in different tissues and different developmental stages, suggesting potential functional divergences among family members (Figure 4).

**Figure 5:**
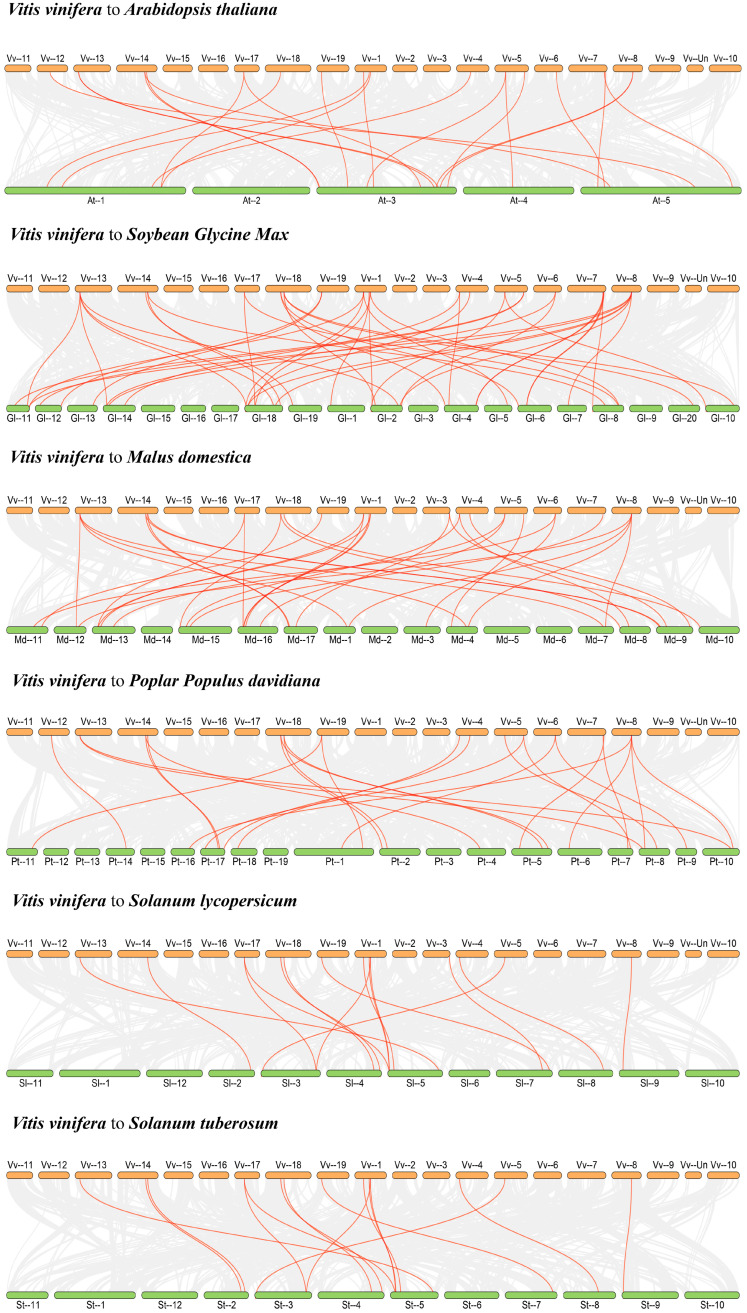
Inter-group collinearity analysis of *RCC1s* between *V. vinifera* and six other plant species: *A. thaliana*, *G. max*, *M. domestica*, *P. davidiana*, *S. lycopersicum*, and *S. tuberosum*. Red lines indicate syntenic *RCC1* gene pairs between *V. vinifera* and each reference genome.

To further evaluate the evolutionary pattern of VvRCC1 genes across different species, we employed interspecific collinearity analysis with RCC1 proteins from *Malus domestica*, *Arabidopsis thaliana*, *Populus davidiana*, *Solanum tuberosum*, *Solanum lycopersicum*, and *Glycine max* (Figure 5). Numerous collinear gene pairs were identified between *Vitis vinifera* and these species, indicating that RCC1 genes are evolutionarily conserved in major angiosperm groups. The findings reveal that *Vitis vinifera* had the highest abundance of conserved homologous pairs with *Malus domestica* and *Glycine max*. reflecting a strong evolutionary relationship with these species. There were fewer lines that were collinear with *A. thaliana* and *S. tuberosum*. A number of *VvRCC1* genes such as *VvRCC1-4*, *VvRCC1-6*, *VvRCC1-13*, and *VvRCC1-18* had several syntenic links to other species suggesting these genes are evolutionarily conserved and functional. The widespread collinearity across different species indicates that both segmental duplication and chromosomal rearrangements have greatly contributed to the expansion and diversification of the RCC1 gene family in grapevine. The conserved roles of RCC1 in plant taxa are also illustrated by these synteny patterns.

### 2.5. Gene Expression Across Grape Tissues and Developmental Stages

Analysis of the expression levels of *VvRCC1* genes in different tissues across 54 grapevine tissues and developmental stages ranging from seedling to fruit and seed development (Table S2). Analysis shows that these genes have specific places in time and space where they act at different growth phases. It is observed that *VvRCC1-2* (*Vitvi01g00915*) and *VvRCC1-6* (*Vitvi04g00838*) are highly expressed in most tissues and specifically in tendril, bud and rachis in grapes. Probably, it takes part in growth and stress mechanisms. Genes such as *VvRCC1-12* (*Vitvi07g01964*) and *VvRCC1-16* (*Vitvi12g00779*) exhibit high expression in buds and rachis, respectively, suggesting their involvement in flower initiation and fruit set. Genes *VvRCC1-1* (*Vitvi01g00591*) and *VvRCC1-5* (*Vitvi04g00292*) are moderately expressed in various tissues, indicating a possible involvement in maintenance or general cellular processes during the grapevine’s development.

**Figure 6:**
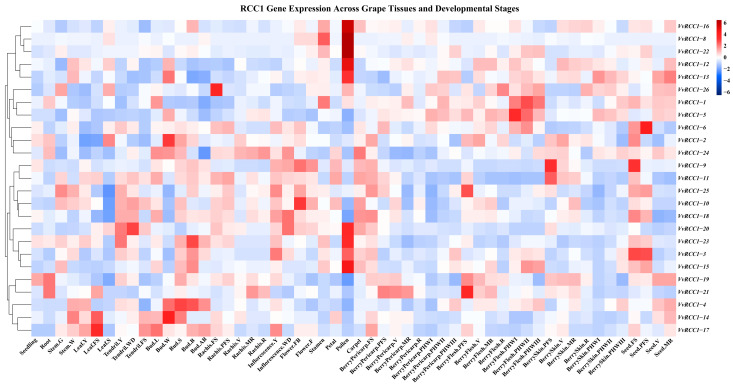
Heatmap of the grapevine *VvRCC1* genes expression across grape tissues and developmental stages. The expression levels of *VvRCC1s* are indicated by the color intensity. Red and blue boxes indicate high and low expression levels, respectively. On the left side of the heatmap, a phylogenetic tree was constructed by hierarchical clustering of the expression profiles of the *VvRCC1s* in the 54 samples, with the full names shown in Table S2.

Also, there are a few genes with low expression in a majority of tissues like *VvRCC1-6* (*Vitvi04g00838*) and *VvRCC1-3* (*Vitvi01g00977*), which may be repressed or regulated in particular developmental stages. Meanwhile, high expressions of genes like *VvRCC1-11* (*Vitvi07g01923*) during flowering and berry pericarp development suggest their involvement in reproductive development and maturity of fruit. Clusters of genes that show similar expression patterns are also revealed in the heatmap. Those involved in reproductive stages, such as *VvRCC1-2* (*Vitvi01g00915*), *VvRCC1-14* (*Vitvi08g02205*), and *VvRCC1-18* (*Vitvi13g01429*), may act in a synergistic manner. Ultimately, the expression data indicated the diverse involvements of *VvRCC1* genes in grapevine development and stress response giving a comprehensive idea of the functional diversification of these genes (Figure 6).

### 2.6. Expression Dynamics of VvRCC1 Genes Under Salt, Drought, and Heat Stress

To investigate the role of *VvRCC1* genes in grapevine responses to abiotic stresses, we examined their expression profiles under salt, drought, and heat stress using qRT-PCR at multiple time points. The expression of ten representative *VvRCC1* genes was measured following exposure to each stress, with sampling conducted at 0, 12, 24, 36, and 48 h for salt and drought stress, and at 0, 0.5, 1, 2, 4, and 6 h for heat stress.

#### 2.6.1. Salt Stress Response

**Figure 7:**
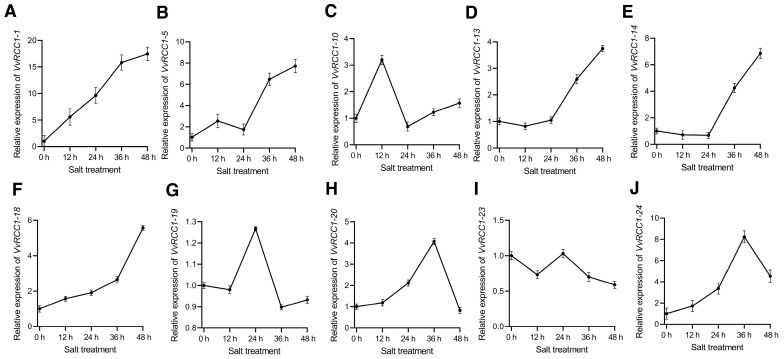
Expression profiles of selected *VvRCC1* genes under salinity stress. Relative expression levels of (**A**) *VvRCC1-1*, (**B**) *VvRCC1-5*, (**C**) *VvRCC1-10*, (**D**) *VvRCC1-13*, (**E**) *VvRCC1-14*, (**F**) *VvRCC1-18*, (**G**) *VvRCC1-19*, (**H**) *VvRCC1-20*, (**I**) *VvRCC1-23*, and (**J**) *VvRCC1-24* were determined by qRT-PCR at 0, 12, 24, 36, and 48 h after drought treatment. Data represent the mean values of three independent biological replicates, and vertical bars indicate the standard error of the mean.

Under salt stress, *VvRCC1* genes exhibited distinct and time-dependent expression patterns, indicative of their potential roles in stress adaptation. *VvRCC1-1* showed the most pronounced and persistent induction, with expression steadily increasing from 12 to 48 h, peaking at a 17-fold increase compared to control, suggesting a critical role in long-term adaptation to salinity stress (Figure 7A). *VvRCC1-5* was moderately induced, with significant upregulation at 36 and 48 h, indicating its involvement in the later stages of salt stress (Figure 7B). In contrast, *VvRCC1-10* exhibited transient upregulation at 12 h, followed by a decrease at 24 h, suggesting its role in early salinity stress perception rather than prolonged adaptation (Figure 7C). *VvRCC1-13* and *VvRCC1-14* showed late-stage induction, with peak expression at 36 and 48 h, implying their involvement in the delayed response to salt stress (Figure 7D,E). Similarly, *VvRCC1-18* displayed sustained induction, reaching its highest expression at 48 h, indicating prolonged involvement in salt stress modulation (Figure 7F).

In contrast, *VvRCC1-19* showed weak expression, with minimal variation during treatment, suggesting a limited role in salt stress response (Figure 7G). *VvRCC1-20* and *VvRCC1-24* exhibited moderate and intermediate responses, with *VvRCC1-20* peaking at 36 h and *VvRCC1-24* reaching its highest expression at 36 h, suggesting their involvement in the mid-phase of salt stress (Figure 7H,J). Lastly, *VvRCC1-23* was predominantly downregulated, with slight recovery at 24 h but further suppression at 36 and 48 h, indicating negative regulation by salt stress (Figure 7I).

#### 2.6.2. Drought Stress Response

**Figure 8:**
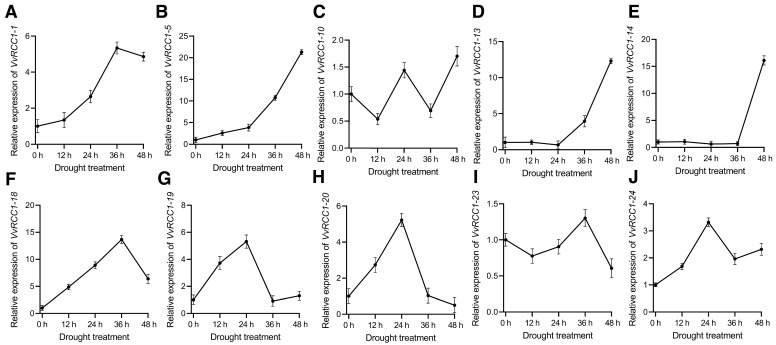
Expression profiles of selected *VvRCC1* genes under drought stress. Relative expression levels (**A**) *VvRCC1-1*, (**B**) *VvRCC1-5*, (**C**) *VvRCC1-10*, (**D**) *VvRCC1-13*, (**E**) *VvRCC1-14*, (**F**) *VvRCC1-18*, (**G**) *VvRCC1-19*, (**H**) *VvRCC1-20*, (**I**) *VvRCC1-23*, and (**J**) *VvRCC1-24* were determined by qRT-PCR at 0, 12, 24, 36, and 48 h after drought treatment. Data represent the mean values of three independent biological replicates, and vertical bars indicate the standard error of the mean.

Exposure to drought stress also resulted in gene-specific expression profiles. *VvRCC1-1* exhibited a progressive increase in expression from 12 to 36 h, with a slight decrease at 48 h, suggesting its involvement in the intermediate phase of drought adaptation (Figure 8A). *VvRCC1-5* displayed the most consistent and pronounced upregulation, peaking at 48 h, indicating its central role in sustained drought response (Figure 8B). *VvRCC1-10* showed fluctuating expression, with minimal induction at 12 h, followed by intermediate levels at 24 h and decreased expression at 36 h, suggesting its temporal involvement in drought stress signaling (Figure 8C). *VvRCC1-13* and *VvRCC1-14* were strongly induced at 36 and 48 h, indicating their potential role in the later stages of drought stress, possibly in cellular protection or stress-adaptive signaling (Figure 8D,E). *VvRCC1-18* was transiently upregulated between 12 and 36 h, with reduced expression at 48 h, pointing to a role in the mid-phase of drought response (Figure 8F). *VvRCC1-19* and *VvRCC1-20* exhibited early and transient induction, peaking at 12 and 24 h, followed by a decrease, suggesting their role in early drought perception and signaling (Figure 8G,H). *VvRCC1-23* showed minimal variation with slight upregulation at 36 h, while *VvRCC1-24* demonstrated moderate induction at 24 h, followed by a partial decline, suggesting their roles in intermediate-stage drought response (Figure 8I,J).

#### 2.6.3. Heat Stress Response

**Figure 9:**
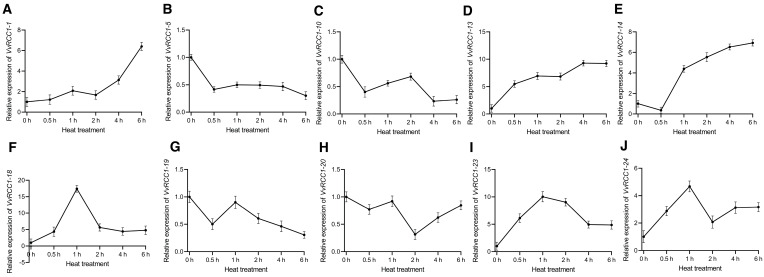
Expression profiles of different *VvRCC1* genes regulated upon exposure to heat stress. Relative expression levels of (**A**) *VvRCC1-1*, (**B**) *VvRCC1-5*, (**C**) *VvRCC1-10*, (**D**) *VvRCC1-13*, (**E**) *VvRCC1-14*, (**F**) *VvRCC1-18*, (**G**) *VvRCC1-19*, (**H**) *VvRCC1-20*, (**I**) *VvRCC1-23*, and (**J**) *VvRCC1-24* were analyzed by qRT-PCR at 0, 0.5, 1, 2, 4, and 6 h after heat treatment. Data represent the mean values of three independent biological replicates, and vertical bars indicate the standard error of the mean.

The response of *VvRCC1* genes to heat stress was characterized by distinct temporal regulation patterns. *VvRCC1-1* displayed a progressive increase in expression, peaking at 6 h with a six-fold increase compared to control, indicating its delayed and sustained involvement in heat stress response (Figure 9A). *VvRCC1-13* and *VvRCC1-14* showed significant early induction, with peak expression at 4 and 6 h, respectively, suggesting their role as major heat-responsive genes (Figure 9D,E). *VvRCC1-18* exhibited transient induction at 0.5 h, followed by repression at later time points, suggesting its involvement in early heat stress detection (Figure 9F). *VvRCC1-23* displayed rapid induction at 1 h, with a subsequent decline from 2 to 6 h, indicating its role in the early response to heat stress (Figure 9I). *VvRCC1-24* showed peak expression at 1 h, followed by suppression at 2 h, and a subsequent increase from 4 to 6 h, suggesting its involvement in rapid heat stress perception (Figure 9J). In contrast, *VvRCC1-5* and *VvRCC1-10* were downregulated throughout the treatment, with VvRCC1-5 showing reduced expression at all time points, while *VvRCC1-10* displayed partial recovery at 1 and 2 h but suppressed expression at later time points (Figure 9B,C). *VvRCC1-19* and *VvRCC1-20* exhibited suppressed expression throughout the heat stress period, with *VvRCC1-19* showing minimal recovery at 1 h and *VvRCC1-20* showing slight recovery at 1 h but declining thereafter (Figure 9G,H).

### 2.7. UV-B-Induced Nuclear Translocation of VvUVR8

**Figure 10:**
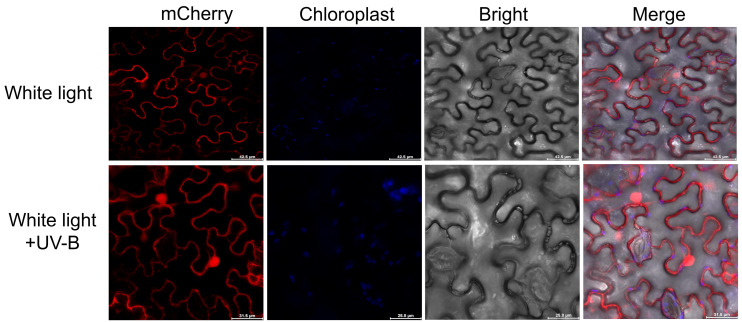
UV-B-induced nuclear accumulation of UVR8. Infiltrated tobacco leaves were treated with white light (−UV-B) or white light supplemented with UV-B (+UV-B) for 24 h. Bar, 42.5 μm.

*VvRCC1-17* (*Vitvi13g00350*) has been identified as the UV-B photoreceptor UVR8 homolog via NCBI BLAST, which plays a central role in perceiving UV-B radiation and initiating downstream UV-B responsive signaling pathways in grapevine. To clarify the functional action site of the protein, subcellular localization analysis was first performed. The subcellular localization of the VvUVR8 protein was assessed using an OE-VvUVR8-mCherry fusion protein, expressed in the epidermal cells of *Nicotiana benthamiana* leaves. The fusion protein was tagged with mCherry to visualize its localization under fluorescence microscopy. The cells were analyzed under two light conditions: white light and white light + UV-B. As shown in Figure 10, mCherry fluorescence was detected in both the cytosol and nucleus of the tobacco leaf epidermal cells in white light. We then analyzed whether UV-B would induce the nuclear accumulation of the UVR8-mCherry fusion protein, and found that UV-B induced its accumulation in the nucleus (Figure 10). There was a tremendous shift observed in the localization of *VvUVR8*-mCherry fusion protein due to UV-B light, suggesting that exposure to UV-B induces the accumulation of the *VvUVR8* proteins in the nucleus.

### 2.8. VvUVR8 Enhances Anthocyanin Accumulation Under UV-B Treatment in Grape

**Figure 11:**
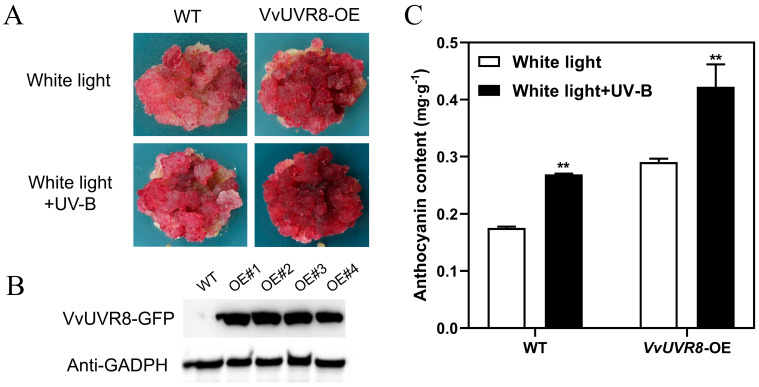
Regulation of anthocyanin biosynthesis by *VvUVR8*. (**A**) Phenotypes of WT and *VvUVR8* transgenic grape calli grown under white or UV-B light; (**B**) Western blot quantification of VvUVR8-GFP; (**C**) Anthocyanin content of WT and *VvUVR8* transgenic grape calli. All data are means ± SD (*n* = 3 biological replicates) and determined by two-way ANOVA and Dunnett’s multiple comparison test. ** represents significant differences between test and control groups (** *p* < 0.01).

To explore the role of *VvUVR8* in regulating anthocyanin biosynthesis in grapevine, we generated stable *VvUVR8* overexpression (OE-VvUVR8) calli via *Agrobacterium*-mediated transformation. The transformation was carried out using the OE-*VvUVR8*-GFP construct, which was selected for the presence of the GFP marker gene. After several cycles of growth on selective medium, stable transgenic grapevine calli were successfully obtained (Figure 11A). The Western blot analysis showed a robust expression of VvUVR8-GFP in the transgenic calli, with a clear band corresponding to the GFPtag, confirming the stable expression of the transgene across multiple independent overexpression (OE)lines. The Western blot analysis showed a robust expression of *VvUVR8*-GFP in the transgenic calli, with a clear band corresponding to the GFP tag, confirming the stable expression of the transgene across multiple independent overexpression (OE) lines (Figure 11B). These data established the successful generation of stable OE-VvUVR8 calli for subsequent analysis.

The results revealed that the overexpression of UVR8 promotes anthocyanin accumulation in grapes (Figure 11A). Meanwhile, UV-B treatment also induced anthocyanin accumulation in wild-type (WT) grape plants. Moreover, UV-B treatment of UVR8-overexpressing transgenic lines further enhanced this effect with a significantly higher degree of promotion. This indicated that grape UVR8 participates in the UV-B-mediated promotion of anthocyanin accumulation. Figure 11C presents the quantitative determination of anthocyanin content corresponding to the phenotypic results in Figure 11A, which further verifies and supports the above conclusion.

## 3. Discussion

The RCC1 (Regulator of Chromosome Condensation 1) family represents a conserved and multifunctional group of nuclear regulatory proteins that coordinate essential cellular processes such as nucleocytoplasmic transport, chromatin organization, and cell-cycle progression [14,15]. In plants, RCC1-domain proteins have acquired additional functional layers, linking environmental perception to developmental programs and stress adaptation [5,8]. Despite the functional significance of RCC1 proteins in model plants, systematic studies of this family in woody perennials, including grapevine (*Vitis vinifera*), have been lacking. Our genome-wide analysis identified 26 *VvRCC1* genes, distributed across 13 chromosomes, with non-uniform localization and evidence of segmental duplication, suggesting both evolutionary expansion and functional diversification within the grapevine RCC1 family. Structural analyses revealed conservation of core RCC1 domains alongside variations in exon-intron organization, motif composition, and physicochemical properties, implying potential specialization of individual family members for distinct cellular and environmental roles. These observations are consistent with studies in other plant species, in which duplicated RCC1 genes have undergone neofunctionalization or sub-functionalization to adapt to environmental pressures [16,17].

Expression profiling revealed that certain VvRCC1 genes are broadly expressed across vegetative and reproductive tissues, suggesting roles in fundamental cellular processes such as genome maintenance, cell division, and chromatin remodeling. In contrast, other members exhibited tissue- or stage-specific expression, particularly during active growth and reproductive development, indicating specialized regulatory functions. The distinct spatiotemporal patterns reflect sub-functionalization following segmental duplication, broadly expressed members maintain the ancestral Ran-GTPase and chromatin functions required for general nucleocytoplasmic transport across all tissues, whereas tissue-specific members have acquired specialized roles in reproductive organ development and stage-specific chromatin remodeling. Similar patterns have been documented in *Arabidopsis thaliana* and *Artemisia annua*, where RCC1 proteins contribute to developmental differentiation and organ-specific functions [5]. Furthermore, several plant RCC1-domain proteins, including *AtRUG3*, *MtZR*, and *OsRLR4/OsRCC1-15*, are known to influence root architecture, meristem activity, and organ growth [18,19,20], supporting the notion that specific VvRCC1 genes may regulate grapevine growth and organ development.

Our study demonstrates that VvRCC1 genes exhibit stress-specific transcriptional regulation under salinity, drought, and heat stress, highlighting their potential integration into complex signaling networks. Under salinity stress, *VvRCC1-1*, *VvRCC1-5*, *VvRCC1-13*, *VvRCC1-14*, and *VvRCC1-18* showed persistent or delayed induction, suggesting their involvement in sustained salt adaptation. In contrast, *VvRCC1-10*, *VvRCC1-20*, and *VvRCC1-24* displayed transient or intermediate responses, whereas *VvRCC1-23* was largely suppressed. This differential responsiveness reflects structural and phylogenetic partitioning within the family: members retaining complete RCC1 β-propeller motifs (e.g., *VvRCC1-1*, *-5*, *-13*, *-14*) exhibit sustained induction consistent with conserved GEF activity required for long-term nuclear transport, whereas structurally divergent or reduced-motif members (e.g., *VvRCC1-10*, *-23*) show transient or suppressed expression, indicating neofunctionalization toward stress perception or feedback modulation. Similar stress-specific transcriptional patterns of RCC1 genes have been observed in cotton, rice, maize, and *Arabidopsis*, supporting a conserved role of RCC1-domain proteins in abiotic stress signaling [8,16,21]. Notably, overexpression of RCC1 homologs such as SaRCC1 in *Arabidopsis* can reduce salt tolerance, emphasizing that RCC1-dependent stress regulation is context-dependent and may involve complex feedback with upstream signaling pathways including ABA-dependent responses, DREB/CBF transcription factors, heat-shock factors, WRKY/MYB regulators, and ROS-mediated signaling [22].

Under drought stress, the transcriptional patterns further highlight the modular nature of RCC1 gene function. *VvRCC1-5* showed the strongest and most sustained induction, whereas *VvRCC1-13* and *VvRCC1-14* were predominantly upregulated during later phases, suggesting their role in prolonged adaptation. Early- or mid-phase induction of *VvRCC1-19*, *VvRCC1-20*, and *VvRCC1-24* suggests potential roles in rapid drought perception or transient stress responses, whereas *VvRCC1-10* and *VvRCC1-23* exhibited low expression, likely reflecting basal or housekeeping activity. These results underscore that stress responses in grapevine are temporally and spatially orchestrated, with different RCC1 genes activated at distinct phases to optimize survival and maintain growth under water-limiting conditions. Similar drought-responsive patterns have been reported in rice and maize, further suggesting evolutionary conservation of RCC1 function in stress adaptation [4,16,23].

Heat stress induced unique transcriptional dynamics within the VvRCC1 family. *VvRCC1-1*, *VvRCC1-13*, and *VvRCC1-14* showed delayed yet persistent upregulation, indicating their involvement in late-phase heat adaptation, whereas *VvRCC1-18*, *VvRCC1-23*, and *VvRCC1-24* were predominantly expressed during the initial stress phase. Conversely, *VvRCC1-5*, *VvRCC1-10*, *VvRCC1-19*, and *VvRCC1-20* were suppressed or displayed variable expression patterns, illustrating the nuanced stress-specific regulatory landscape of the RCC1 family. These observations suggest that RCC1 proteins integrate multiple stress cues to fine-tune cellular responses, potentially through interactions with chromatin remodelers, transcription factors, and signaling molecules involved in ROS detoxification, osmotic adjustment, and heat-shock responses [22,24,25].

At the biochemical level, RCC1 proteins operate as chromatin-associated GEFs that generate and maintain the Ran-GTP gradient essential for nucleocytoplasmic transport and cell-cycle progression [14,15]. The chromatin-bound pool of RCC1 catalyzes the conversion of Ran-GDP to Ran-GTP, creating a high nuclear concentration of Ran-GTP that promotes the dissociation of cargo from importin-β transport receptors, thereby enabling the nuclear accumulation of transcription factors, kinases, and chromatin-remodeling proteins [15]. Under abiotic stress conditions, the demand for rapid nuclear import of stress-responsive transcriptional regulators (e.g., DREB/CBF, WRKY, and MYB family proteins) increases substantially, necessitating dynamic modulation of the Ran-GTP gradient and nuclear pore transport capacity [21,22]. The differential transcriptional regulation of *VvRCC1* genes observed under salinity, drought, and heat stress therefore likely reflects an adaptive reprogramming of nucleocytoplasmic transport efficiency and chromatin accessibility, allowing the cell to prioritize the nuclear delivery of protective transcription factors while reconfiguring chromatin architecture for stress-responsive gene expression [16,21].

Our localization studies demonstrate that *VvUVR8* undergoes rapid nuclear translocation upon UV-B exposure. This light-induced nuclear accumulation facilitates the activation of downstream transcriptional programs. Functional assays revealed that OE-VvUVR8 transgenic grape calli accumulate significantly higher levels of anthocyanins, manifesting in visibly enhanced red pigmentation compared to wild-type calli. These results provide direct evidence that RCC1-domain proteins can act as environmental sensors linking external light cues to metabolic outputs, extending the known functional repertoire of plant RCC1 proteins beyond canonical nuclear regulation and stress response [25,26]. These findings illustrate that the grapevine RCC1 family acts as a multifunctional regulatory hub, coordinating growth, development, and environmental responses [27]. Certain members, such as *VvRCC1-1*, *-5*, *-13*, and *-14*, function as integrators of abiotic stress signaling, while *VvRCC1-17* (*UVR8*) bridges light perception and secondary metabolism. This dual role highlights the evolutionary flexibility of RCC1 proteins, allowing them to mediate both cellular homeostasis and adaptive responses to complex environmental stimuli. Importantly, the identification of candidate genes with stress-specific and light-responsive functions provides a foundation for functional validation using overexpression, knockout, or RNAi approaches, and for exploring downstream signaling and interaction networks. While this study focused on anthocyanin accumulation as the quantifiable phenotypic endpoint, UVR8-mediated UV-B signaling activates the phenylpropanoid pathway at the level of shared early biosynthetic enzymes, including chalcone synthase and flavanone 3-hydroxylase, which likely modulates the broader phenolic profile encompassing flavonols and proanthocyanidins. Future metabolomic analyses will be required to determine the full extent of *VvUVR8*-dependent phenolic reprogramming in grapevine. These insights expand the functional landscape of plant RCC1 proteins, highlighting their potential utility in improving stress resilience and crop quality in grapevine.

## 4. Materials and Methods

### 4.1. Plant Materials and Growth Conditions

Young and healthy stems were collected as explants from the grape cultivar Red Globe (*Vitis vinifera* L. cv. Red Globe), which had been maintained through repeated sub-culturing in Woody Plant Medium (WPM) under controlled growth room conditions with temperature 25 ± 2 °C, photoperiod of 16 h light/8 h dark, light intensity of 100 µmol m^−2^ s^−1^, and relative humidity of 60–70%, located in State Key Laboratory for Crop Stress Resistance and High Efficiency Production, Northwest A & F University, Yangling, Shaanxi, China. Plants used for abiotic stress treatments and sample collection were derived exclusively from these in vitro sub-cultured stocks, with no field-grown material used at any stage.

The 1 L media was prepared by using 800 mL distilled water in a beaker, followed by the addition of WPM powder (2.14 g), sucrose (30 g), Agar (7 g), Ca(No_3_)_2_ (0.56 g), 6-BA (0.2 mg/L), and IBA (0.2 mg/L). It was mixed thoroughly, and its final volume was then adjusted to 1 L. Its pH was checked using a pH meter and adjusted to 5.8–5.9. After that, carbon-activated charcoal powder (1.5 g) was added to the whole media and was autoclaved at 121 °C at 15 psi for 40 min. The media was transferred to the jars when cooled down inside a laminar flow unit. After cooling, the young and healthy stem explants were cultured in this medium and kept in the growth room for optimum growth.

Each treatment consisted of three replicates, with each replicate comprising three tissue-cultured plants bearing an average of 8–10 leaves. For each data point, two leaves per plant were harvested, pooled from the three plants within each replicate (six leaves total per replicate), immediately frozen in liquid nitrogen, and stored at −80 °C until further use. These samples were used for RNA extraction and qRT-PCR analysis (Section 4.9), subcellular localization (Section 4.10), and protein extraction and immunoblots (Section 4.12).

### 4.2. Identification and Retrieval of RCC1 Genes

The reference genome sequence, protein sequences, coding sequences (CDS), and genome annotation file (GFF3) of *Vitis vinifera* were downloaded from Ensembl Plants (http://plants.ensembl.org, version: Release 62; accessed on 1 October 2025). To identify RCC1 family members in grapevine, the Hidden Markov Model (HMM) profile of the RCC1 domain, corresponding to Pfam accession PF00415, was downloaded from the Pfam database. This HMM profile was used as a query to screen the grapevine protein database using HMMER [28]. Candidate RCC1-containing proteins were retrieved based on significant domain hits, and redundant sequences were manually removed. To ensure the reliability of the identified candidates, all putative VvRCC1 proteins were further examined using Pfam and SMART to confirm the presence of an intact RCC1 domain [29,30]. Proteins lacking the conserved RCC1 domain or containing incomplete domain structures were excluded. Finally, 26 non-redundant RCC1 genes were identified in the grapevine genome and retained for subsequent analyses.

### 4.3. Computational Analysis of Physicochemical Properties

The ProtParam tool available at ExPASy server (https://web.expasy.org/protparam/; accessed on 5 October 2025) was used to examine the physicochemical properties of VvRCC1 proteins [31]. Amino acid composition, molecular weight (MW), theoretical isoelectric point (pI), instability index and grand average of hydropathicity (GRAVY) were calculated to evaluate molecular characteristics and predicted stability of protein. Subcellular localization of VvRCC1 proteins was predicted using the Plant-mPLoc server (http://www.csbio.sjtu.edu.cn/bioinf/plant-multi/; accessed on 8 October 2025), which integrates multiple localization signals and sequence-derived features [32].

### 4.4. Conserved Sequence Alignment and Phylogenetic Analysis

To investigate the evolutionary relationships of RCC1 proteins, RCC1 protein sequences from *V. vinifera* and six representative dicotyledonous species, including *Arabidopsis thaliana*, *Glycine max*, *Malus domestica*, *Populus davidiana*, *Solanum lycopersicum*, and *Solanum tuberosum*, were used for phylogenetic analysis. The protein sequence datasets of these species were downloaded from public genome databases, including Ensembl Plants. RCC1-domain-containing proteins were identified using the Hidden Markov Model profile of the RCC1 domain corresponding to Pfam accession PF00415. The HMM profile was used to screen the protein datasets of each species with HMMER, and candidate RCC1 proteins were further verified using Pfam and SMART to confirm the presence of conserved RCC1 domains. Redundant proteins that were not interacting with RCC1 domains was filtered out before further analysis.

MAFFT version 7.520 was used with default parameters to perform the multiple sequence alignment of the RCC1 proteins [33]. The aligned sequences were then used to construct a maximum-likelihood phylogenetic tree using IQ-TREE version 2.2.2.7 [34]. The optimal substitution model, Q.plant+R7, was selected based on model testing. Branch support was evaluated using 1000 ultrafast bootstrap replicates. The phylogenetic tree was visualized and annotated using Interactive Tree of Life (iTOL) version 6 [35].

### 4.5. Chromosomal Distribution, Conserved Motif Identification, and Structure Analysis

Genomic positions of *VvRCC1* genes were extracted from the grapevine GFF3 annotation file and visualized using the GTF/GFF plotting function of TBtools; version, v2.475 [36]. To discover conserved motifs in the VvRCC1 protein family, full-length protein sequences were analyzed using MEME Suite (http://meme-suite.org) [37]. The uppermost limit of motifs was set to ten and other parameters like motif width were kept at default values. Comparison of conserved motifs across phylogenetic subgroups was conducted. Using the Gene Structure View (Advanced) module of TBtools, we analyzed the gene structure (exon–intron organization and motif distribution) based on genome annotation files, protein sequences and MEME motif output.

### 4.6. Synteny and Gene Duplication Analysis

The One-Step MCScanX module implemented in TBtools was used to analyze collinearity and duplicated gene pairs within the *V. vinifera* genome and between grapevine and other plant species. Intraspecific syntenic relationships among *VvRCC1* genes were used to identify WGD/segmental duplication events, whereas tandem duplication was assessed based on the physical proximity of paralogous genes on the same chromosome. According to the MCScanX classification system, WGD/segmental duplicates are inferred from anchor genes located in collinear blocks, tandem duplicates are adjacent paralogs on the same chromosome, proximal duplicates are nearby paralogs separated by several non-homologous genes, and dispersed duplicates are duplicated genes that are neither physically close nor located within conserved syntenic blocks. Transposed duplicated genes were not classified in this study because their reliable identification requires additional pipelines, such as MCScanX-transposed, and comparative analysis with appropriate outgroup genomes. The Advanced Circos and Dual Synteny Plot functions of TBtools were used to visualize intra- and interspecific syntenic relationships [36].

### 4.7. Expression Profiling of VvRCC1 Genes

RNA-sequencing datasets were retrieved from the NCBI Gene Expression Omnibus (GEO). To investigate the gene expression patterns during the development and across different tissues, we utilized high-throughput microarray data from a gene expression atlas encompassing various organs and tissues at different developmental stages [38]. The 54 sample datasets analyzed in this study were obtained from the Gene Expression Omnibus of NCBI (GSE36128). Heatmaps were generated using the pheatmap package in the R software, version R.4.5.2, performing hierarchical clustering using default distance metrics and clustering method.

### 4.8. Abiotic Stress Treatments and Sample Collection

Plants at 6–8 leaf stages were used during this experiment. They were exposed to drought, salinity, and high temperature stress. For drought stress, the plants were treated with 20% PEG-6000 solution, whereas NaCl at the rate of 200 mM was used to induce the salinity stress in the plants. The plants were placed in the growth room and the leaf samples were collected at 0, 12, 24, 36 and 48 h, respectively. For high temperature stress, plants with 6–8 leaf stages were placed inside a growth chamber and the caps of the tissue culture bottles were removed. The light intensity of the growth chamber was set to 12,000 Lx with a relative humidity of 45%. These plants were treated at 45 °C, and the samples were collected at 0, 0.5, 1, 2, 4, and 6 h. All these samples were immediately put in liquid nitrogen and were placed at −80 °C until further use.

### 4.9. RNA Extraction and qRT-PCR Analysis

Primers for the 10 RCC1 genes were designed using the NCBI Primer BLAST tool; Version, 2.17.0 (https://www.ncbi.nlm.nih.gov/tools/primer-blast/; accessed on 8 October 2025). The primers used in this study are provided in Table S3. Total RNA extraction, cDNA synthesis, dilution, and quality of the cDNA were assessed using the protocols from our previous studies. The *VvGAPDH* was used as a control, and the relative expression of the genes was determined using the 2^−ΔΔCT^ method [39].

### 4.10. Subcellular Localization

The pCAMBIA2300-VvUVR8-mCherry construct was introduced into *Agrobacterium tumefaciens* GV3101 strain, and was subsequently mixed with P19 (inhibit gene science) in a 1:1 ratio. The resulting mixed bacterial solution was injected into the abaxial surface of 4-week-old *Nicotiana benthamiana* leaves (3 to 5 leaves from the tip) using a 1 mL syringe (needle removed). After 24 h of darkness followed by 48 h of light, the injected *N. benthamiana* leaves were collected for imaging. Fluorescence signals of mCherry were observed using a Laser Scanning Confocal Microscope (Leica, TCS SP8 SR, Wetzlar, Germany) to determine the distribution location of proteins in the cells. The experiment was repeated three times.

### 4.11. Grape Calli Transformation

The agrobacterial strains harboring pCAMBIA2300-VvUVR8-GFP construct were cultured in 50 mL liquid LB medium until the OD_600_ value reached 0.4–0.6. Bacterial cells were collected by centrifugation at 5000× *g* for 10 min, then resuspended in infiltration buffer (1/2 GS medium supplemented with 20 g·L^−1^ sucrose and 200 μM acetosyringone, AS). The suspension was incubated at 28 °C with shaking at 110 rpm in darkness for 3 h.

Grape calli were immersed in the agrobacterial suspension for 10 min, then blotted dry with sterile filter paper. The infected calli were transferred onto co-cultivation medium and incubated at 23 °C in darkness for 2 d. Subsequently, the calli were rinsed twice with bacteriostatic solution (450 mg L^−1^ Carbendazim + 450 mg L^−1^ Cefotaxime) for 3 min each time, followed by two washes with sterile distilled water. After blotting excess moisture with filter paper, the calli were transferred to selective medium (GS medium supplemented with 200 mg L^−1^ Carbendazim, 200 mg L^−1^ Cefotaxime and 60 mg L^−1^ Kanamycin) for screening. The surviving resistant grape calli were subcultured and maintained as independent lines.

### 4.12. Protein Extraction and Immunoblots

Total proteins were isolated from the collected samples using an extraction buffer composed of 50 mM Tris (pH 7.6), 150 mM NaCl, 10% glycerol, 5 mM MgCl_2_, 0.1% NP-40, 1% dithiothreitol (DTT), and 1% protease inhibitor cocktail (purchased from Sigma, Shanghai, China). The extracted proteins were separated by sodium dodecyl sulfate-polyacrylamide gel electrophoresis (SDS-PAGE) and then transferred onto polyvinylidene fluoride (PVDF) membranes following the standard protocol provided by the manufacturer (Bio-Rad, Shanghai, China). A primary antibody against GFP and GADPH (Proteintech, Wuhan, China) was employed for antigen recognition, and a secondary anti-mouse antibody (Abmart, Shanghai, China) was used as recommended for signal amplification. Finally, the target protein signals were visualized using a LumiBest ECL reagent solution kit.

### 4.13. Anthocyanin Content Determination

Transgenic grape calli were rapidly ground into fine powder in a pre-cooled mortar. The powder was spread evenly in a Petri dish, covered with plastic wrap with reserved ventilation holes, and then lyophilized in a vacuum freeze-dryer for subsequent anthocyanin determination. Exactly 0.5 g of lyophilized powder was weighed and transferred into a sterile 50 mL centrifuge tube, followed by the addition of 10 mL of 60% methanol–hydrochloric acid solution. Ultrasonic extraction was performed at 30 °C with 40 kW power for 40 min. The extract was centrifuged at 12,000 rpm for 10 min at 4 °C, and the supernatant was collected. The extraction procedure was repeated three times, and all supernatants were combined. Subsequently, 0.5 mL of the combined extract was transferred into two separate test tubes, to which 4.5 mL of KCl buffer (pH 1.0) and CH_3_COONa·3H_2_O buffer (pH 4.5) were added, respectively. The mixtures were incubated in the dark for 15–20 min. The absorbance values were measured at 520 nm and 700 nm with three biological replicates. The total anthocyanin content was finally calculated and expressed as malvidin-3-O-glucoside equivalent (mg/g FW).

## 5. Conclusions

This study presents the first genome-wide characterization of the RCC1 gene family in *Vitis vinifera*, identifying 26 VvRCC1 genes with diverse phylogenetic, structural, and transcriptional profiles. Conserved RCC1 domains indicate essential regulatory roles, while variation in gene organization and stress-responsive expression patterns suggests functional diversification. Segmental duplication appears to have driven family expansion. qRT-PCR analyses revealed differential regulation under salinity, drought, and heat stress, with *VvRCC1-13* and *VvRCC1-14* showing consistent upregulation across these conditions, identifying them as candidate stress-responsive genes for further investigation. *VvRCC1-1* and *VvRCC1-5* displayed stress-specific induction and repression patterns, indicating finely tuned transcriptional divergence. Mechanistically, *VvRCC1-17* functions as a UV-B photoreceptor (UVR8 homolog), undergoing nuclear accumulation upon UV-B exposure. Overexpression of *VvUVR8* in grape calli enhanced anthocyanin accumulation, further potentiated by UV-B treatment, linking environmental perception to downstream metabolic responses. While this study establishes a genomic and transcriptomic framework for *VvRCC1* genes, it is important to note that functional inferences for most family members are based on expression profiling alone, and direct genetic validation, such as knockout, RNAi, or stable whole-plant transgenic analyses, remains to be performed. Furthermore, *VvUVR8* overexpression and anthocyanin quantification were conducted in callus culture, and confirmation in whole plants is required to fully assess agronomic applicability. These findings provide a foundational genomic resource for viticultural improvement, linking specific *VvRCC1* loci to abiotic stress responses and UV-B signaling. Collectively, this work integrates genomic, transcriptional, and molecular evidence, identifying RCC1 genes as candidate mediators of stress adaptation and UV-B signaling in grapevine, and provides compelling targets for future functional studies aimed at improving resilience and metabolic regulation in perennial fruit crops.

## Figures and Tables

**Table 1:** Genomic, protein, and subcellular properties of *VvRCC1* gene family members.

VvRCC1’s	Gene Name	Chr	Genomic Location	CDS Length (bp)	Protein					Subcellular Location
					Size (aa)	MW (Da)	Gravy	PI	Instability Index	
*VvRCC1-1*	*Vitvi01g00591*	1	6670437–6675258	3490	534	57,715.45	−0.128	6.07	85.13	Nucleus
*VvRCC1-2*	*Vitvi01g00915*	1	10895630–10898793	1191	396	41,965.2	−0.171	6.11	80.68	Nucleus
*VvRCC1-3*	*Vitvi01g00977*	1	11850540–11888230	3144	1047	113,315.098	−0.42	8.41	72.81	Nucleus
*VvRCC1-4*	*Vitvi03g01304*	3	20478502–20490945	1506	434	45,842.19	−0.018	6.39	88.53	Nucleus
*VvRCC1-5*	*Vitvi04g00292*	4	2747775–2750977	2168	466	50,937.89	−0.259	5.12	79.87	Mitochondrion, Nucleus
*VvRCC1-6*	*Vitvi04g00838*	4	10604132–10614117	3460	1082	120,075.32	−0.479	8.69	73.77	Nucleus
*VvRCC1-7*	*Vitvi04g04334*	4	17996934–17997722	789	262	28,938.76	0.039	9.32	90.42	Nucleus
*VvRCC1-8*	*Vitvi05g00753*	5	8161180–8171160	3186	1061	115,907.38	−0.439	8.44	74.25	Nucleus
*VvRCC1-9*	*Vitvi05g01494*	5	22677958–22680738	2781	926	101,820.93	−0.174	6.86	82.22	Nucleus
*VvRCC1-10*	*Vitvi06g01245*	6	16760977–16784949	3324	1107	120,066.95	−0.502	9.17	71.42	Nucleus
*VvRCC1-11*	*Vitvi07g01923*	7	27281115–27293344	1915	445	47,723.02	−0.366	5.57	75.06	Nucleus
*VvRCC1-12*	*Vitvi07g01964*	7	27937267–27971814	1763	420	44,898.65	−0.133	6.73	74.6	Nucleus
*VvRCC1-13*	*Vitvi08g00173*	8	3331342–3343443	4246	1076	116,898.71	−0.403	9.11	82.36	Nucleus
*VvRCC1-14*	*Vitvi08g02205*	8	15096631–15102257	1464	487	51,490.07	−0.239	6.28	76.1	Nucleus
*VvRCC1-15*	*Vitvi12g00662*	12	8736715–8737275	561	186	19,713.48	0.115	6.02	87.42	Cell wall, Nucleus
*VvRCC1-16*	*Vitvi12g00779*	12	9928489–9944442	1299	432	45,229.33	0.056	5.73	93.84	Nucleus
*VvRCC1-17*	*Vitvi13g00350*	13	3640236–3647906	1841	484	50,692.96	−0.176	5.74	74.94	Nucleus
*VvRCC1-18*	*Vitvi13g01429*	13	22156658–22204217	4135	1104	119,174.55	−0.416	9.07	74.16	Nucleus
*VvRCC1-19*	*Vitvi14g01227*	14	21535618–21545618	1877	395	42,313.72	−0.328	6.67	80.99	Nucleus
*VvRCC1-20*	*Vitvi14g01340*	14	23016482–23030740	1982	478	51,580.88	−0.29	5.74	78.18	Nucleus
*VvRCC1-21*	*Vitvi17g04104*	17	5381456–5382019	564	187	19,726.95	0.333	9.56	106.52	Nucleus
*VvRCC1-22*	*Vitvi17g04156*	17	7280898–7323732	2577	823	89,496.71	−0.395	8.63	73.15	Nucleus
*VvRCC1-23*	*Vitvi18g01069*	18	11707146–11718406	2301	542	58,240.19	−0.649	8.77	65.46	Nucleus
*VvRCC1-24*	*Vitvi18g01277*	18	14121658–14133818	4335	1129	122,282.54	−0.429	8.57	77.28	Nucleus
*VvRCC1-25*	*Vitvi19g00251*	19	3491356–3498750	2717	500	54,397.68	−0.094	7.92	81.66	Nucleus
*VvRCC1-26*	*Vitvi19g01652*	19	22464454–22465494	810	269	29,437.66	−0.07	6.75	81.86	Nucleus

## Data Availability

Data will be made available on request.

## Supplementary Materials

The following supporting information can be downloaded at: https://www.mdpi.com/article/10.3390/plants15111709/s1, Table S1: Protein sequences from species used for phylogenetic tree construction; Table S2: Full and abbreviation names for 54 different organs and developmental stages in grapevine; Table S3: Primers used for qRT-PCR of VvRCC1 genes.
